# Determinants of food insecurity among adults residing in peri-urban municipal settings in Flanders, Belgium

**DOI:** 10.1186/s12889-024-19389-7

**Published:** 2024-07-29

**Authors:** Yasemin Inaç, Suzannah D’Hooghe, Karin De Ridder, Sarah Dury, Nico Van de Weghe, Eva M. De Clercq, Delfien Van Dyck, Benedicte Deforche, Stefanie Vandevijvere

**Affiliations:** 1 Sciensano, Department of Epidemiology and Public Health, Brussels, Belgium; 2 Sciensano, Department of Chemical and Physical Health Risks, Brussels, Belgium; 3 Faculty of Medicine and Health Sciences, Department of Public Health and Primary Care, Ghent University, Ghent, Belgium; 4 Faculty of Psychology and Educational Sciences, Vrije Universiteit Brussel, Adult Educational Sciences, Brussels, Belgium; 5 Faculty of Sciences, Department of Geography, Ghent University, Ghent, Belgium; 6 Faculty of Physical Education and Physiotherapy, Department of Movement and Sport Sciences, Vrije Universiteit Brussel, Brussels, Belgium; 7 Faculty of Medicine and Health Sciences, Department of Movement and Sports Sciences, Ghent University, Ghent, Belgium

**Keywords:** Food security, High-income countries, Food environment, Social environment, Health inequities

## Abstract

Food insecurity is a global public health issue associated with noncommunicable diseases. Individual factors are strongly associated with food insecurity, but there is limited literature on the broader impact of both the social and food environments on food insecurity in non-English speaking European countries, given that the research was predominantly conducted in Anglophone settings. In addition, these studies have mostly been conducted in urban areas. Therefore, this study aimed to identify the main determinants of food insecurity among adults living in peri-urban areas in Flanders, Belgium. Data on socio-demographics, neighborhood social cohesion, social isolation, and perceived food environments were collected from 567 adults through a self-administered questionnaire, and objective data on the food environment were obtained through (commercial) databases on food outlets. Food insecurity was measured using the USDA Household Food Security Survey Module. Multivariable logistic regression models revealed that lower socioeconomic status (OR14.11,95%CI:4.72;61.11), reasonable (OR4.16,95%CI: 2.11;8.47) to poor and very poor (OR6.54,95%CI: 2.11;8.47) subjective health status, and living in private (OR7.01, 95% CI:3.0;17.0) or government-assisted (OR6.32,95%CI: 3.13;13.26) rental housing significantly increased the odds of food insecurity. Additionally, residing in a neighborhood with low (OR2.64, 95% CI:1.13;6.26) to medium (OR2.45,95% CI:1.21;5.11) social cohesion, having a neutral opinion (OR4.12,95%CI:1.51;11.54) about the availability of fruit and vegetables in one's neighborhood, and having an opinion that fruit and vegetable prices are too expensive (OR5.43,95% CI 2.26;14.4) significantly increased the odds of experiencing food insecurity. This study underscores the need for policies that consider factors related to social and food environments, in addition to individual factors, to effectively address food insecurity.

**Supplementary Information:**

The online version contains supplementary material available at 10.1186/s12889-024-19389-7.

## Introduction

Food insecurity in high-income countries has been linked to several determinants, which can be broadly categorized into individual-, social-, and food environment determinants [1].

Individual determinants such as lower educational attainment, lower income, unemployment, renting a home, and single parenthood have been clearly linked to food insecurity through previous studies [2, 3]. However, individual determinants do not fully explain why some populations experience food insecurity whereas others do not [4]. Understanding the role of individual determinants is crucial, but it is equally important to consider broader determinants to gain a comprehensive understanding of food insecurity.

Social determinants such as social cohesion and social isolation, also seem to play a significant role in experiencing food insecurity. Social cohesion, i.e. the degree of connectivity and solidarity among residents of a neighborhood [5], has been shown to influence food insecurity. For example, a study in the United States (US) reported that households exposed to low levels of social cohesion were significantly more likely to experience food insecurity [6]. Similarly, a study from the US by Nebbitt et al. (2016) reported that neighborhood social cohesion and a sense of belonging buffered the experience of food insecurity among residents of government social housing [7]. Although, the link between social cohesion and food insecurity appears multifaceted. Whereby, current literature suggests that less social cohesion in a neighborhood may threaten the community's capacity to develop social and/or economic support among members, which may increase food insecurity [8]. Social isolation, i.e. one’s lack of interaction with the broader community, is another social factor that has been shown to influence food insecurity. For example, social isolation was demonstrated to be a characteristic of food-insecure households, but also a determinant that facilitated the continuation and escalation of food insecurity within the household [7, 9].

The food environment, i.e. the physical, economic, political and socio-cultural context in which consumers engage with the food system, has also been previously linked with food insecurity [10, 11]. Previous studies have demonstrated that an obesogenic food environment (i.e., an environment that promotes high-energy intake and sedentary behavior) is associated with an increased likelihood of experiencing food insecurity. Certain populations, such as the elderly, people residing in rural locations, and people with lower socioeconomic status, are more dependent on their local food environment due to transportation issues and lower incomes [12, 13, 14]. Consequently, they are more vulnerable to the adverse effects of unhealthy food environments.

There is evidence that the food environment is place-depended and differs between urban, peri-urban and rural areas. The food environment in peri-urban areas (i.e., zones of transition between urban and rural land, located between the outer limits of urban and regional centers and the rural environment [15]) has both urban and rural environmental characteristics and may pose a unique challenge in accessing food, distinct from those in purely urban or rural settings [16]. However, to the best of our knowledge, research on the determinants of food insecurity in peri-urban areas is lacking.

In Belgium, research on food insecurity is limited. Even though according to Eurostat’s 2022 food poverty indicator,, 4.1% of Belgian households reported that they could not afford to buy a meal containing fish, meat, or a vegetarian alternative every second day [17]. Food aid figures provide an estimate of this issue. In 2022, about 3% of the adult Belgian population used the services of the Belgian Federation of Food Banks at least once during the last year [18]. Vandevijvere et al. (2021) assessed food insecurity during the COVID-19 pandemic using three separate indicators and found that 10.4% of respondents often or sometimes feared food shortages, 5.0% were often or sometimes short of food, and 10.3% often or sometimes could not afford a healthy diet in the last 30 days [19]. Although the study by Vandevijvere et al. was conducted during the COVID-19 pandemic, it indicates that food insecurity is a significant issue in Belgium.

In summary, despite food insecurity being a significant issue in Belgium, few studies have address this phenomenon. Those that have primarily focused on urban areas, leaving peri-urban and rural areas understudied. Identifying and managing populations at risk of food insecurity is crucial due to its detrimental effects on public health. Recognizing the importance of this issue, access to adequate food is enshrined in the Universal Declaration of Human Rights [20, 21]. Therefore, this study aimed to investigate the individual-, social-, and food environment determinants of food insecurity among adults residing in two peri-urban municipalities in Flanders, Belgium.

## Methods

Data were obtained as part of the CIVISANO-project in two peri-urban municipalities in the Flemish-speaking region of Belgium:,- Duffel and Herselt. The full details of the project can be found elsewhere [22]. The study was conducted in accordance with the recommendations of the Belgian Data Protection Authority and was approved by the Medical Ethics Committee of Ghent University Hospital (BC-248 09260). Each respondent signed an informed consent form prior to participation.

### Sampling procedure

Data collection took place between May and November 2021, as part of the mixed-methods CIVISANO-project. The sampling strategy for the project conservatively aimed to recruit approximately 254 respondents, based on a sample size calculation, which was conducted to calculate the sample size for the quantitative arm of the project. Full details on the sample size calculation for the project can be found elsewhere (D’Hooghe et al., 2022). In the CIVISANO-project, an overrepresentation of respondents with lower socioeconomic status was intended. Therefore, respondents were recruited primarily through active recruitment, which is similar to time-location-based sampling. Whereby, locations such as food banks/distributions, local (social) organizations, remedial schools neighborhoods with a higher concentration of government-assisted social housing and private rentals in which people with a lower socioeconomic status were overrepresented were compiled into a list and these locations were randomly visited by volunteers during the recruitment period. The volunteers offered respondents the option to fill in the questionnaire themselves using a tablet, or guided the respondents through the questionnaire using an interview approach. Simultaneously, other sampling strategies were also utilized, for example positing information and QR-codes to fill in the questionnaire on traditional (local) paper-based- and social media and making the questionnaire available in local places, which were not visited as part of the active recruitment, such as general practitioners offices, pharmacies and libraries.

### Food insecurity

Food insecurity was measured at the household level using three screening questions adapted from the screener version of the United States Department of Agriculture (USDA) Household Food Security Survey Module [23]. The first two questions focused on overall food insecurity (“We worried whether the food would run out before we got money to buy more,” and “The food that we bought did not last and we did not have money to get more"). The third question focused on food insecurity related to "healthy" foods ("We did not have enough money to buy fresh fruit- and vegetables"). Respondents were asked to indicate whether they had experienced the situations described in the statements in the past 30 days. Response options included: "never," "sometimes," and "always." Respondents were considered food insecure if they answered "sometimes" or "always" to at least one of the three questions.

### Determinants

Data on individual, social, and food environment determinants were collected through a self-administered questionnaire and objective GIS-based data.

### Individual determinants

Individual determinants included sociodemographic and lifestyle factors such as socioeconomic status, subjective health status, household composition, housing tenure, and transportation to food outlets.

To determine the respondents’ socioeconomic status, the variable socioeconomic status (SES) was created, with two categories: lower socioeconomic status (LSES) or higher socioeconomic status (HSES). The classification was based on meeting at least one of the following criteria: (a) low level of education (no tertiary level), (b) no current paid employment, (c) net household income below the national minimum income (i.e. €1625.72 gross per person per month in 2021), taking into account household size, (d) perceived financial difficulties (= difficult to very difficult to make ends meet on a monthly basis), or (e) low perceived socioeconomic status (less than or equal to five on the MacArthur scale (Adler et al., 2000)). This approach was based on an extensive review of the literature, which resulted in the inclusion of both objective and subjective determinants of SES, and is consistent with previous studies of composite measures of SES [24, 25].

The subjective health status variable was determined by asking, “How would you rate your general health?”. Responses were measured on a five-point Likert scale ranging from “very poor” to “very good.” These were later merged into three categories, namely “good/very good”, “reasonable” and “poor /very poor”. For the variable household composition, respondents were classified as having no underage children (if no children under the age of 18 were present in the household), single parents (if there was only one person over the age of 18 in the household and at least one child under the age of 18), or as a multi-parent household (if there were at least two people over the age of 18 and one child under the age of 18 in the household).

To create the variable housing tenure, respondents were asked, ‘Which statement about your home is correct?’. Answer categories included home owner, private rental, government-assisted rental, and none of the above (e.g., living with adult children, usufruct use of a property, etc.). The transport to food outlets variable was created by categorizing transport to food outlets as active transport (walking and/or cycling) or motorized transport (car or public transport use). Respondents were classified as using active transport if they selected cycling or walking as a form of transportation they used to reach food outlets, or motorized transport if they selected driving a car or using public transport as their primary mode of transportation.

### Social environment determinants

The determinants of the social environment include social cohesion and social isolation. The social cohesion variable was based on eight statements derived from the Flemish version of the SPOTLIGHT questionnaire [26]. Respondents were given eight statements about interactions in their neighborhood (e.g., in my neighborhood people take care of each other, in my neighborhood people help each other, etc.). Based on the responses to these statements, social cohesion was classified as high (i.e., positive answer to at least six statements), medium (i.e., positive response to four or five statements), or low (i.e., less than four statements with a positive response) in the neighborhood. This classification was based on a review of the literature, primarily the SPOTLIGHT study on neighborhood social capital [27] and after discussions with members of the Civisano research group. Regarding social isolation, the number of friends and family members living nearby was used as a proxy variable. Respondents were asked to indicate the number of friends and family living nearby on a five-point scale ranging from “many” to “none.” This resulted in the creation of the social isolation variable, which was treated as a continuous variable.

### Food environment determinants

Food environment determinants were divided into perceived and objective dimensions. Two perceived dimensions were included: the perceived availability and price of fresh fruits and vegetables in the neighborhood. All perceived variables were based on two statements in the questionnaire, which were taken from the NEMS-P questionnaire [28]. The statements included: “Fresh fruits and vegetables are easily available in my neighborhood” and “Fresh fruits and vegetables are cheap to buy in my neighborhood”. Answer categories ranged from “Completely disagree” to “Completely agree” on a 5-point Likert scale. These were later merged into three categories, namely “agree/strongly agree”, “neutral” and “strongly disagree/disagree”.

Regarding the objective food environment, the absolute density and proximity of healthy food outlets were assessed for all the respondents. To calculate both metrics, the respondents were asked to localize the intersection nearest to their home address when filling in the questionnaire. The nearest intersection was used instead of the home address to protect the privacy of the respondents. Each respondent’s location was linked to a database of healthy food outlets in the municipalities. Data on these outlets were obtained from the Locatus 2020 database, supplemented with data on short-chain initiatives such as ‘*Recht Van Bij De Boer’*, local (farmers) markets, farm stores, and community gardens [29]. Healthy food outlets were defined as outlets that primarily sell healthy food, such as greengrocers, fishmongers, and farmers’ markets. This definition was based on the opinion of an expert committee consisting of food policy experts and nutritionists from Flanders. More information on the classification of food outlets can be found in the study by Smets et al. [30]. In line with current practices supermarkets were classified as healthy food outlets [31]. Buffers of 500m and 1000m around the residence were used to calculate the healthy outlet density. These buffer sizes were chosen based on previous studies conducted internationally and in Ghent (Belgium) as part of the ‘International Physical Activity and Environment Network' (IPEN) which recommends the use of street network buffers of 500m and 1000m around respondents’ residences to develop a standardized spatial definition of a 'neighborhood which could be used to compare results across countries [32, 33]. In addition, two different buffer sizes were used to account for potential variations in the food environment and travel behaviors of respondents to food outlets [34]. Proximity to healthy food outlets was defined as the shortest road network distance in meters to the nearest healthy outlet, and was calculated for each respondent along the street network using ArcGIS Pro.

### Covariates

Directed acyclic graphs (DAGs) were constructed using DAGitty software for each (potential) association between the independent variables and the dependent variable (i.e., food insecurity) to select covariates and reflect on the structure between the variables, see supplementary file [35]. Based on this, for each association under study, covariates were selected based on the hypothesized association between the variables under study. This was based on an extensive literature review. The selection of covariates for which each model was adjusted is displayed using footnotes in Table 2. In addition, the respondents’ age and gender identities were included as covariates in all analyses. Age was measured in years and rounded off to the nearest whole number. Respondents’ gender identity was utilized as “male,” “female” or “prefer not to answer.”

### Data analysis

#### Study population

Descriptive statistics were used to describe the characteristics of the total sample and the food insecure respondents. Continuous variables were presented as means and standard deviations (SD). Categorical variables were presented as frequencies.

#### Treatment of missing data

Multiple imputation by chained equations (MICE) was used to estimate the missing values for the independent- and dependent variables as well as the covariates. This technique relied on available values for all variables collected in this study. This method followed the approach outlines by Van Buuren and Groothuis-Oudshoorn (2011), and was implemented using the MICE package in RStudio [36]. The pooled estimates of the five imputed datasets were used for the remainder of the analysis. The amount of missingness for each variable is listed in Table 1.

**Table 1:** Individual, social,- and food environment related characteristics of the total sample and by food security group. Respondents in the CIVISANO-survey, Flanders, 2021

	Total sample *n* = 567		Food insecure *n* = 99		Food secure *n* = 468	
	*n*	%	*n*	%	*n*	%
**Individual determinants**						
Gender identity						
Female	366	64.6	67	67.7	299	63.8
Male	193	34.0	32	32.3	161	34.4
Other	4	0.7	0	0	4	0.9
Missing	4	0.7	0	0	4	0.9
Age (mean ± SD)	46.0 (11.1)		46.7 (11.1)		45.9 (11.0)	
Socio-economic status						
LSES	327	52.7	96	96.7	231	49.4
HSES	240	42.3	3	3.3	237	50.6
Subjective health status						
Very bad/bad	53	9.4	29	29.3	24	5.1
Reasonable	209	36.9	47	47.5	162	34.6
Good/very good	303	53.4	23	23.2	280	59.8
Missing	2	0.3	0	0	2	0.4
Household composition						
Single parenthood						
Yes	56	9.9	16	16.2	40	8.5
Multiple parents						
Yes	200	35.3	23	23.2	177	37.8
Underage children						
No	311	54.9	60	60.6	251	53.6
Housing type						
Homeowner	426	75.1	33	33.3	393	84.0
Private rental	44	7.8	23	23.2	21	4.5
Social housing	70	12.3	37	37.4	33	7.1
Other	21	3.7	5	5.1	16	3.4
Missing	6	1.1	1	1.0	5	1.2
Transport to food outlets						
Active transport						
Yes	316	55.7	64	64.6	252	53.8
Motorized						
Yes	479	84.5	67	67.7	412	88.0
**Social environmental determinants**						
Social cohesion						
High-level	231	40.7	18	18.2	213	45.5
Medium-level	106	18.7	21	21.2	85	18.2
Low-level	223	39.3	58	58.6	165	35.3
Missing	7	1.2	2	2.0	5	1.1
Number of family/friends nearby						
Multiple/many	218	38.5	21	21.2	197	42.1
Little/some	264	46.5	53	23.5	211	45.1
None	85	15.0	25	25.3	60	12.8
Missing	0	0	0	0	0	0
**Food environmental determinants**						
Objective food environment						
Density of healthy food outlets in the 500m buffer (mean ± SD)	1.4 (1.9)		1.9 (2.1)		1.3 (1.8)	
Density of healthy food outlets in the 1000m buffer (mean ± SD)	1.9 (2.1)		1.9 (2.2)		2.2 (2.0)	
Proximity of healthy food outlets in meters (mean ± SD)	766.2 (761.6)		656.6 (638.7)		788.2 (782.7)	
Perceived food environment						
In my neighborhood, fresh fruits and vegetables are easily available						
Strongly disagree/disagree	33	5.8	12	12.1	21	4.5
Neutral	34	6.0	17	17.2	17	3.6
Agree/strongly agree	496	87.5	69	69.7	427	91.2
Missing	4	0.7	1	1.0	3	0.6
In my neighborhood, fresh fruit and vegetables are cheap						
Strongly disagree/disagree	217	38.7	61	61.6	156	33.8
Neutral	197	35.1	29	29.3	168	36.4
Agree/strongly agree	147	26.2	9	9.1	138	29.9

#### Association between individual, social and food environment determinants and food insecurity

To assess potential associations between food insecurity and individual, social environment, and food environment determinants, multivariable logistic regression analysies was used, adjusted for multiple covariates depending on the hypothesized associations between the variables.

Variables that were statistically significant in the separate models, as shown in Table 2, were included in the overall model, which is shown in Table 3. The overall model was adjusted for age and gender. In addition, multicollinearity was examined using variance inflation factors (VIF) for each separate model and the overall model. The VIF did not find evidence of multicollinearity in any of the models, with values for most determinants approaching two and/or one, and some approximating between two and three. None of the determinants were near or above five, suggesting no multicollinearity. Statistical tests were two-sided, and differences or associations were considered statistically significant at *p* < 0.05. All analyses were performed using RStudio software.

**Table 2:** Logistic regression analysis of the association between individual, social- and food environmental characteristics and food insecurity. Respondents in the CIVISANO-survey, Flanders, 2021

Determinants	Adjusted OR	95%	Nagelkerke pseudo R^2^
**Individual characteristics**			
Socioeconomic status^a^			0.38
LSES	32.46***	(11.59;135.74)	
HSES	Reference		
Subjective health status^b^			0.38
Very poor/poor	9.44***	(4.57;20.14)	
Reasonable	4.28***	(2.38;7.86)	
Good/very good	Reference		
Housing tenure^c^			0.40
Homeowner	Reference		
Private rental	7.42***	(3.58;15.68)	
Social housing	6.50***	(3.55;12.10)	
Other	2.43	(0.72;7.22)	
Household composition^d^			0.27
Single parent	0.89	(0.44;1.71)	
Multiple parents	Reference		
Transport to food outlets^e^			0.31
Active transport			
Yes	1.05	(0.61;1.83)	
No	Reference		
Motorized transport			
Yes	0.33*	(0.18;0.61)	
No	Reference		
**Social environment characteristics**			
Social cohesion^f^			0.43
High	Reference		
Medium	7.42***	(3.58;15.68)	
Low	6.51***	(3.55;12.10)	
Social isolation^g^	1.30***	(1.02;1.6)	0.28
**Objective food environment characteristics**			
Density of healthy food outlets in the 500m buffer^h^	1.11	(0.96;1.28)	0.28
Density of healthy food outlets in the 1000m buffer^i^	1.01	(0.89;1.14)	0.27
Proximity to healthy food outlets^j^	1.0	(1.0;1.001)	0.28
In my neighborhood, fresh fruits and vegetables are easily available^k^			0.31
Strongly disagree/disagree	1.89	(-0.18;1.41)	
Neutral	4.66***	(0.72;1.41)	
Agree/strongly agree	Reference		
In my neighborhood, fresh fruit and vegetables are cheap^l^			0.34
Strongly disagree/disagree	5.03***	(2.43;11.53)	
Neutral	2.64*	(1.21;6.28)	
Agree/strongly disagree	Reference		

^a^Adjusted for subjective health status, age and gender identity

^b^^−^^d^Adjusted for SES, age and gender identity

^e^Adjusted for proximity to healthy food outlets, SES, age and gender identity

^f^Adjusted for housing tenure, SES, age and gender identity

^g^Adjusted for social cohesion, housing tenure, SES, age and gender identity

^h^^−^^i^Adjusted for proximity to healthy food outlets, SES, age and gender identity

^j^Adjusted for density of healthy food outlets 500m/1000m, SES, age and gender identity

^k^Adjusted for density of healthy food outlets 500m/1000m, proximity to healthy food outlets, SES, age and gender identity

^l^Adjusted for money spend on food per week, SES, age and gender identity

^*^p < 0.05, ***p < 0.001

**Table 3:** Overall model encompassing statistically significant characteristics from the individual, social- and food environmental models. Respondents in the CIVISANO-survey, Flanders, 2021

Determinants	Adjusted OR	95%	Marginal effects	95%	Nagelkerke pseudo R^2^
**Overall model**^**a**^					0.56
Socioeconomic status					
LSES	14.11***	(4.72;61.11)	0.19***	(0.13;0.24)	
HSES	Reference				
Subjective health status					
Very poor/poor	6.54***	(2.79;15.87)	0.16***	(0.08; 0.25)	
Reasonable	4.16***	(2.11;8.47)	0.12***	(0.06;0.17)	
Good/very good	Reference				
Housing tenure					
Homeowner	Reference				
Private rental	7.01***	(3.0;17.0)	0.18***	(0.08;0.29)	
Government assisted social housing	6.32***	(3.1;13.26)	0.16***	(0.08;0.24)	
Other	2.79	(0.62;11.50)	0.07	(-0.07;0.21)	
Transport to food outlets					
Motorized transport					
Yes	0.59	(0.29;1.22)	-0.06	(-0.12;0.01)	
No	Reference				
Social cohesion					
High social cohesion	Reference				
Medium social cohesion	2.45*	(1.21;5.11)	0.09*	(0.02;0.16)	
Low social cohesion	2.64*	(1.13;6.26)	0.18***	(0.08;0.29)	
Social isolation	1.27	(0.98;1.64)	0.02	(-0.01;0.04)	
In my neighborhood, fresh fruits and vegetables are easily available					
Strongly disagree/disagree	0.63	(0.23;1.61)	-0.04	(-0.11;0.02)	
Neutral	4.12***	(1.51;11.54)	0.12*	(0.02;0.23)	
Agree/strongly agree	Reference				
In my neighborhood, fresh fruit and vegetables are cheap					
Strongly disagree/disagree	5.43***	(2.26;14.4)	0.13***	(0.06;0.19)	
Neutral	2.20	(0.87;6.0)	0.05	(-0.02;0.11)	
Agree/strongly agree	Reference				

^a^Adjusted for age and gender identity, *p < 0.05, ***p < 0.001

## Results

### Study population

In total, data was collected from 567 participants, of whom 99 (17.5%) were food insecure and 468 (82.5%) were food secure, as shown in Table 1. The mean age of the participants was 46.0 years, with food insecure participants being slightly older (46.7 years) than food secure participants (45.9 years). The majority of participants were female (64.6%).

7.2. Association between individual, social and food environment determinants and food insecurity.

Table 2 shows the results of the univariable logistic regression analysis. Regarding the individual characteristics, socioeconomic status was found to be the primary factor influencing food security status. Respondents with LSES were 32.46 (95%CI:11.59;135.74) times more likely to experience food insecurity than those with HSES. In addition, those who classified their health as being reasonable were 4.28 (95%CI:2.38;7.86) times more likely to experience food insecurity than those who rated their health as good to very good, while respondents who rated their health as poor to very poor were 9.44 (95%CI:4.57;20.14) more likely to experience food insecurity. Respondents living in government assisted social housing were 6.50 times (95%CI:3.55;12.10) more likely to experience food insecurity compared to homeowners. The odds ratio for respondents living in a privately rented home was 7.42 (95%CI:3.58;15.68) indicating that they were 7.42 times more likely to experience food insecurity compared to homeowners. Household composition (i.e. single or multiple parents in the household) was not observed to be statistically significantly associated with food security status. Motorized transport to food outlets was observed to decrease the odds for experiencing food insecurity (OR:0.33, 95%CI: 0.18;0.61).

In regard to, the social environment determinants, both measures increased the odds for respondents to experience food insecurity. Respondents who reported a low-level of social cohesion in their neighborhoods were 6.51 (95%CI:3.55;12.10) times more likely to experience food insecurity than respondents who reported high levels of social cohesion in their neighborhoods. Respondents who reported medium levels of social cohesion were 7.42 (95%CI:3.58;15.68) times more likely to be food insecure. Social isolation was observed to increase the odds of experiencing food insecurity by 1.36 (95% CI:1.1;1.7) for respondents who reported higher levels of social isolation.

Two things were observed for the food environment determinants. None of the determinants from the objective dimension of the food environment (i.e. density of healthy food outlets in the 500m and 1000m buffers and the proximity to healthy food outlets) were statistically significantly associated with food insecurity. Both dimensions of the perceived food environment showed statistically significant associations. However, not all levels of the perceived dimensions were statistically significant. Respondents with a neutral opinion about the availability of fruit-and vegetables were 4.69 (95%CI:2.1;10.86) more likely to experience food insecurity. Having a negative opinion about the availability of fruit-and vegetables in the neighborhood was not found to be statistically significant. Respondents with a neutral opinion about the price of fruit-and vegetables in their neighborhood were 2.64 (95%CI:1.21;6.28) more likely to experience food insecurity. While respondents with a negative opinion about the price, were 5.03 (95%CI:2.43;11.53) more likely to be food insecure compared to respondents with a positive opinion about the price of fruit-and vegetables.

Table 2 also included the Nagelkerke pseudo R^2^ values, demonstrating that the total variance in food insecurity explained by the univariable models ranged between 0.27 and 0.43. Indicating that the total variance in food insecurity was explained between 27.0% and 43.0% by the separate univariable models.

The model depicted in Table 3 shows the overall multivariable model. The second and third column report the adjusted odds ratio (OR) and subsequent confidence intervals (CI) and indicate that that LSES (OR:14.11,95%CI:4.72;61.11).perceiving one’s health as reasonable (OR:4.16,95%CI:2.11;8.47), poor to very poor (OR:6.54, 95%2.79;15.87) and living in government assisted (OR:6.32, 95%CI: 3.1;12.26) and private rental housing (OR:7.01, 95% 3.0;17.0) increased the likelihood of experiencing food insecurity. Additionally, low (OR: 2.64, 95%CI: 1.13;6.26) to medium (OR:2.45,95%CI:1.12;5.11) social cohesion increased respondents’ likelihood of experiencing food insecurity. Having a neutral opinion (OR:4.12,95%CI:1.51;11.54) about the price of fruit-and vegetables also increased the likelihood of experiencing food insecurity. Similarly, disagreeing with the statement that fruit- and vegetables were cheap to buy in the neighborhood increased the likelihood of experiencing food insecurity (OR:5.43,95%CI:2.26;14.4).” Table 3 also included the Nagelkerke pseudo R^2^ value for the overall multivariable model. The overall model showed a Nagelkerke pseudo R^2^ of 0.56 indicating that 56.0% of the total variance in food insecurity was explained by the included variables. Next to the Nagelkerke pseudo R^2^, Table 3 also includes the marginal effects of the variables included in the overall model. The marginal effects ranged between -0.06 and 0.19 indicating that some variables have a higher predictability for food insecurity compared to others. For the individual determinants, socioeconomic status was shown though have the greatest predictability for food insecurity, whereby low socioeconomic status resulted in a 19.0% increase of food insecurity (95%CI 0.13;0.24), followed by residing in a privately rented home which resulted in a 18.0% increase of food insecurity (95%CI 0.08;0.29), while residing in government-assisted social housing resulted in a 16.0% increase of food insecurity (95%CI 0.08;0.24). Poor to very poor subjective health status also resulted in a 16.0% increase of food insecurity (95%CI 0.08; 0.25), while reporting one’s health as reasonable appeared to increase food insecurity by 12.0% (95%CI 0.06;0.17). For the social environment, low neighborhood social cohesion appeared to increase food insecurity by 18.0% (95%CI 0.08;0.29), while medium neighborhood social cohesion resulted in a 9.0% (95%CI 0.02;0.16) increase of food insecurity. For the food environment determinants, having a neutral opinion about the availability of fruit and vegetables in the neighborhood appeared to increase food insecurity by 12.0% (95%CI 0.02;0.23). While, disagreeing or strongly disagreeing with the statement that fruit and vegetables were cheap in the neighborhood resulted in a 13.0% increase of food insecurity (95%CI 0.06;0.19).

## Discussion

This study investigated the association between individual, social, and food environment determinants with food insecurity among adults in two peri-urban Flemish municipalities. It showed that more than one-sixth of the sample was food insecure. The full models showed that food insecurity was associated with lower socioeconomic status, lower subjective health status, residing in government social housing or a privately rented home, and having a negative perception of the availability and price of fresh fruits and vegetables in the neighborhood (independent of recorded availability and pricing).

Individual determinants therefore seem to play a major role in determining food insecurity; however, determinants from more domains were associated in explaining food insecurity. However, most studies on the determinants of food insecurity have focused on individual determinants. Consistent with our findings, multiple studies have shown that food insecurity rates are higher in households with lower socioeconomic status [37, 38]. Our finding that residing in government social housing or a privately rented home is associated with food insecurity is also reflected in the current literature [39, 40]. Evidence from countries such as the United States indicates that renters spend a disproportionate amount of their income on housing compared to homeowners [41]. This may result in households choosing between multiple basic needs when faced with financial limitations. Qualitative research on people's lived experiences of food insecurity indicates that households are more likely to pay rent first, stating that ‘the rent eats first,’ resulting in a limited budget for food and other expenses [42]. Surprisingly, we did not observe a statistically significant association between household composition (i.e. single parenthood) and food insecurity. Even though this has been reported in previous studies [43–45]. This may be due to the low amount of single parent led household in our sample (*n* = 56) in comparison to household led by multiple parents or without children present. In addition, rating one’s health from reasonable to very poor was also found to be an individual determinant of food insecurity. This is consistent with other studies from high-income countries, which determined that food insecurity is associated with an increased risk of non-communicable health conditions, such as diabetes and cardiovascular conditions [46–48]. However, the direction of the association remains unclear, and it may be that the association is bidirectional, in which poor health leads to restricted finances and, subsequently, to an increased risk for food insecurity, and poor access to healthy food might worsen health.

Social determinants, specifically social cohesion, were associated with the respondents' food security status. Lower neighborhood social cohesion increased the likelihood of experiencing food insecurity. This is consistent with the findings from studies conducted in Anglophone settings. Evidence from the US has shown that social cohesion is related to the magnitude of experiencing food insecurity and that a higher level of social cohesion can partly mitigate food insecurity [5, 6]. Studies from the United Kingdom also suggest that social cohesion has a protective effect on food insecurity; however, these studies have focused on social cohesion as part of larger food network projects such as community agriculture [49, 50]. Besides this evidence, the influence of social cohesion on food insecurity has rarely been studied in a European context. This topic warrants further research, as there are significant differences between the US and Europe in terms of social welfare, ethnic make-up of the population, and food environment, which could have a major effect on the applicability of the study results. Our findings suggest that a higher level of social cohesion positively affects food security in a European setting. In contrast, a recent study in the Netherlands that assessed social cohesion as part of a livability index, reported that only the housing tenure part of the index was associated with food insecurity, rather than overall social cohesion [38]. This may be due to differences in social cohesion between the Netherlands and Belgium. Additionally, the study of Van der Velde et al. was focused on disadvantaged urban neighborhoods, while this study focused on peri-urban neighborhoods. It is likely that these types of neighborhoods may differ in terms of population density, demographics, and build-up, which could influence social cohesion. As these factors could influence neighborhood social cohesion, it would be interesting for future research to study different types of European neighborhoods in relation to social cohesion and food insecurity.

Food environment determinants, specifically the objective ones, were not associated with food insecurity. However, perceived food environment determinants such as the perceived availability and affordability of fruits and vegetables were found to be associated with food insecurity. This indicates that respondents’ perceptions of the prices of fruits and vegetables in their neighborhood might influence food insecurity more than the objective density and proximity to food outlets in their neighborhoods. This is striking because previous work on food insecurity has hypothesized that access to food outlets, often measured by density and proximity metrics, directly influences the food security status. However, a recent Dutch study by Van de Velde et al. that assessed the influence of exposure to fast food outlets on household food insecurity and diet quality found no association between exposure to food outlets and food insecurity [51]. This may be due to the relationship between experiencing food insecurity and receiving food aid. This is because the respondents in the van der Velde study were primarily receiving food aid. People who receive the majority of their food through food aid may have a different perception of their food environment compared to people who purchase the majority of their food in outlets, because they may interact differently with their objective food environment.

The results of this study indicate that although individual determinants are important predictors of the probability of being food insecure, social and environmental determinants also play a role in the experience of food insecurity. By focusing on these determinants rather than individual ones alone, food insecurity is placed in a larger socio-ecological context in which the broader environment plays a role in shaping individuals’ behavior towards their food security status. Since the majority of the population interacts with their social and food environments on a daily basis, this is a primary target for studies on the determinants of food insecurity. In addition, interventions and/or policies geared towards ameliorating food insecurity should be developed. These factors should consider the social and environmental dimensions related to food insecurity.

### Strengths and limitations

A strength of this study is that it is one of the first to assess food insecurity in a Belgian context. To the best of our knowledge, only two studies on food insecurity have been conducted in Belgium [19, 52]. Therefore, this study fills this gap by assessing the determinants of food insecurity in a non-English speaking European country. A second strength is the inclusion of a broad range of determinants spanning the individual, social, and food environmental domains, which has enabled the extensive exploration of determinants related to food insecurity. However, not all determinants of food insecurity could be assessed in this study due to gaps in the questionnaire design. For example, ethnicity, which previous studies have found to have a clear association with food insecurity, could not be included in this study because only nationality could be assessed. This could have led to respondents being classified as having Belgian nationality while potential non-Belgian ethnicity was not taken into account, for example, with second- and third-generation migrants of Turkish and Moroccan ethnicity. An additional strength is the inclusion of many people from underserved communities. This ensured a more comprehensive understanding of food insecurity, as these communities often experience higher rates of food insecurity. However, because recruitment was conducted using an active approach, in which people were encouraged to participate in the study, there may have been selection bias.

A limitation of this study is its cross-sectional design, which makes it impossible to draw causal inferences about factors related to food insecurity. Possible reverse causations might have occurred regarding factors associated with food insecurity, but these were not determinants of food insecurity (e.g., subjective health status). Therefore, caution should be exercised when interpreting the results. Another limitation was that food insecurity among children was not specifically assessed. However, it is known that experiencing food insecurity as a child can have detrimental effects on one’s health throughout life. To the best of our knowledge, childhood food insecurity has not yet been studied in Belgium, and we therefore recommend future studies on this topic.

## Conclusion

In conclusion, our study showed that individual, social, and food environmental determinants influence the risk of food insecurity. Individual determinants, including socioeconomic status, subjective health status, and housing type, emerged as the strongest determinants to increase the likelihood of experiencing food insecurity. This underscores the need for public policies that address the underlying health inequities that underlie food insecurity. Nevertheless, our findings highlight the influence of social and food environment determinants on food insecurity. In addition to individual characteristics, factors such as social cohesion, perceived availability, and the cost of fruits and vegetables were identified as determinants of food insecurity. This underlines the multifaceted nature of food insecurity and shows the importance of community-focused interventions aimed at amelioration through social support networks to enhance neighborhood social cohesion. These findings underscore the inclusion of perceptions in both research and interventions on food security. Interventions should take a holistic approach, addressing both community-aspects and individual vulnerabilities. Future research should broaden the scope and include perceived dimensions when exploring food insecurity. Longitudinal studies are also recommended, which could shed light on the causal relationship between individual, social, and food environment determinants and the onset and duration of food insecurity are recommended.

### Supplementary Information

Supplementary Material 1.

## Data Availability

The datasets generated and analyzed during the current study are not publicly available due to privacy reasons regarding the vulnerable respondents of this study. But might be available from the corresponding author on reasonable request and with permission of Sciensano.

## Abbreviations

CI: Confidence interval
FI: Food insecurity
FV: Fruit-and vegetables
HSES: People with higher socioeconomic status
LSES: People with lower socioeconomic status
SD: Standard deviation
SES: Socioeconomic status
OR: Odds ratio
