# Supplementary material for: Determinants of food insecurity among adults residing in peri-urban municipal settings in Flanders, Belgium
Source: BMC Public Health. 2024 Jul 29;24:2034. doi: 10.1186/s12889-024-19389-7 (PMC11287922; doi:10.1186/s12889-024-19389-7)

Supplementary file - Determinants of food insecurity among adults residing in peri-urban municipal settings in Flanders, Belgium

Yasemin Inaç^1,,2,4,5,7^, Suzannah D'Hooghe^1,3,4^, Karin De Ridder^1^, Sarah Dury^4^, Nico Van de Weghe^5^, Eva M. De Clercq^2^, Delfien Van Dyck^7^, Benedicte Deforche^3,6^, Stefanie Vandevijvere^1^

**^1^**Sciensano, Department of Epidemiology and Public Health, Belgium

^2^Sciensano, Department of Chemical and Physical Health Risks, Belgium

**^3^**Ghent University, Faculty of Medicine and Health Sciences, Department of Public Health and Primary Care, Belgium

**^4^**Vrije Universiteit Brussel, Faculty of Psychology and Educational Sciences, Adult Educational Sciences, Belgium

**^5^**Ghent University, Faculty of Sciences, Department of Geography, Belgium

^6^Vrije Universiteit Brussel, Faculty of Physical Education and Physiotherapy, Department of Movement and Sport Sciences, Belgium

**^7^**Ghent University, Faculty of Medicine and Health Sciences, Department of Movement and Sports Sciences, Belgium

*Corresponding author: Yasemin Inaç – Yasemin.Inac@Sciensano.be

Directed Acyclic Graphs for the models included in table 2:

- Model 1:
  - Outcome: food insecurity
  - Predictor: socioeconomic status
  - Adjusted for subjective health status, age and gender identity


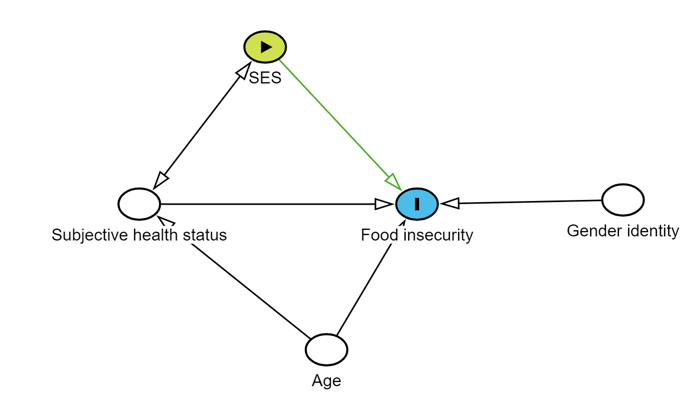


- Model 2:
  - Outcome: food insecurity
  - Predictor: subjective health status
  - Adjusted for socioeconomic status, age and gender identity


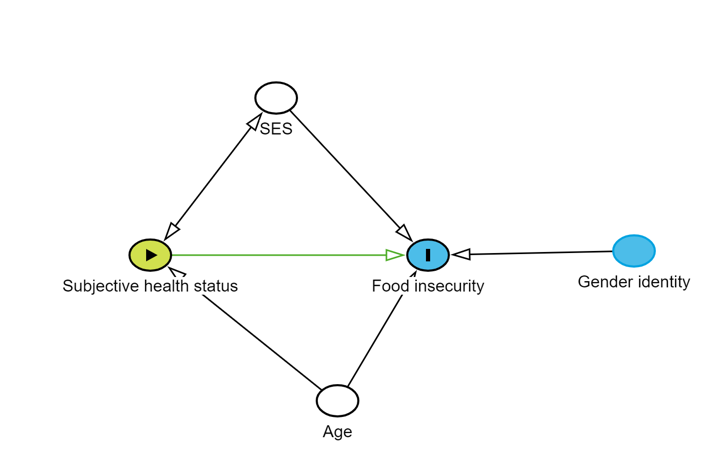


- Model 3:
  - Outcome: food insecurity
  - Predictor: housing tenure
  - Adjusted for socioeconomic status, age and gender identity


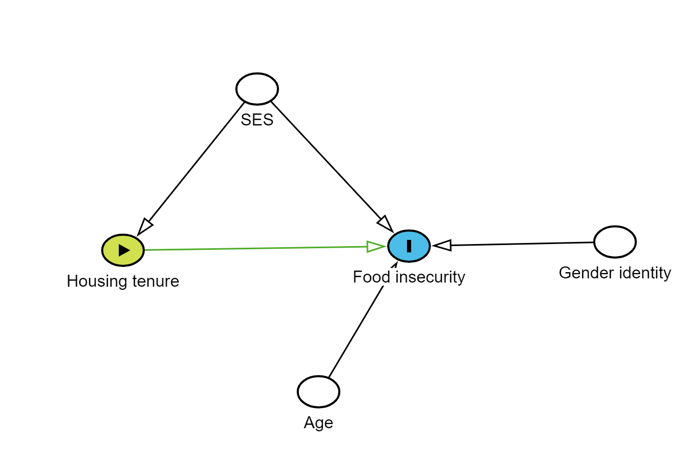


- Model 4 :
  - Outcome : food insecurity
  - Predictor : household composition
  - Adjusted for SES, age and gender identity


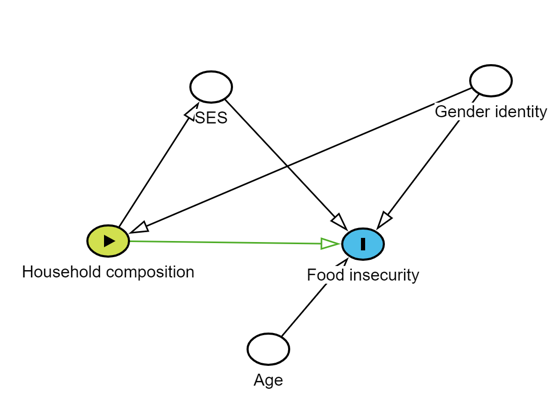


- Model 5:
  - Outcome: food insecurity
  - Predictor: transport to food outlets
  - Adjusted for proximity to healthy food outlets, SES, age and gender identity


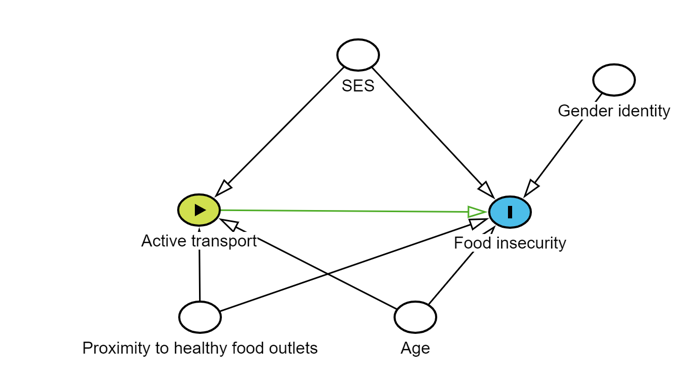


- Model 6:
  - Outcome: food insecurity
  - Predictor: neighborhood social cohesion
  - Adjusted for housing tenure, SES, age and gender identity


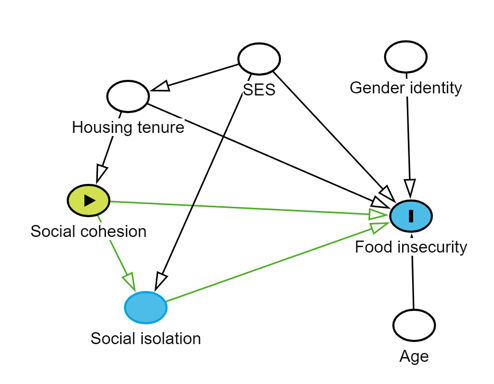


- Model 7:
  - Outcome: food insecurity
  - Predictor: neighborhood social cohesion
  - Adjusted for social cohesion, housing tenure, SES, age and gender identity


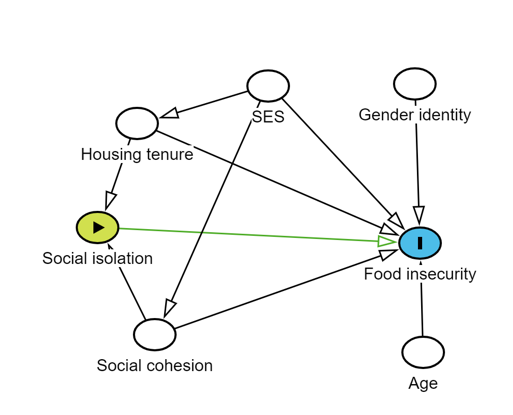


- Model 8:
  - Outcome: food insecurity
  - Predictor: density of healthy food outlets in the 500m buffer
  - Adjusted for proximity to healthy food outlets, SES, age and gender identity


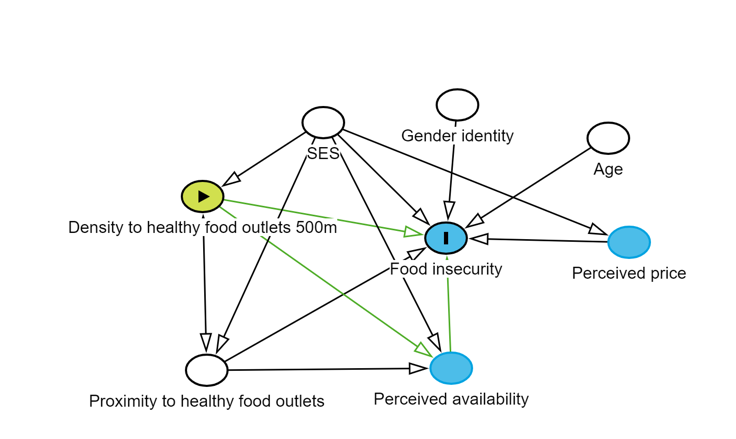


- Model 9:
  - Outcome: food insecurity
  - Predictor: density of healthy food outlets in the 1000m buffer
  - Adjusted for proximity to healthy food outlets, SES, age and gender identity


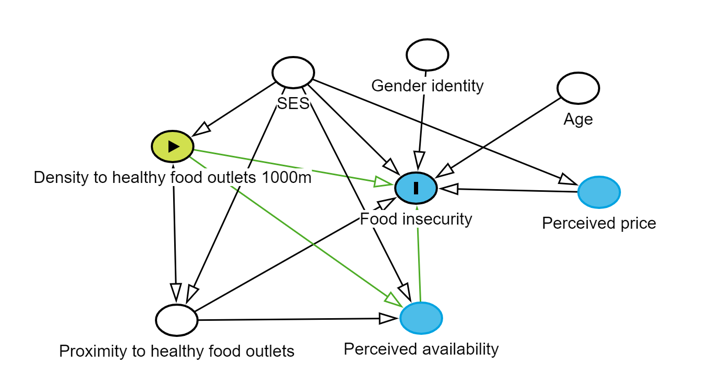


- Model 10:
  - Outcome: food insecurity
  - Predictor: proximity to healthy food outlets
  - Adjusted for density of healthy food outlets in the 500m/1000m buffer, SES, age and gender identity


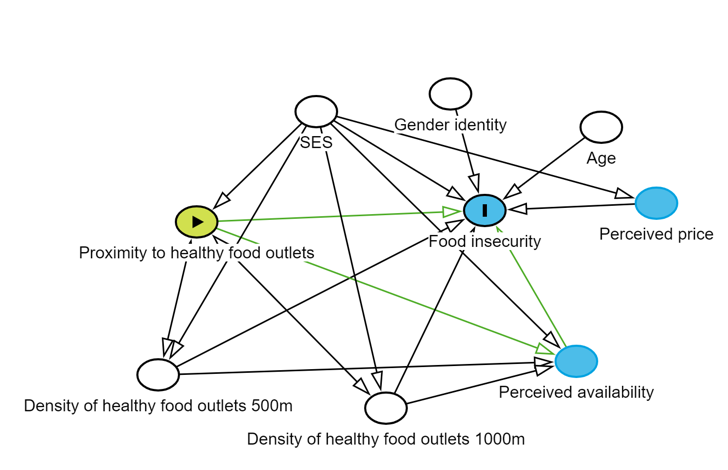


- Model 11:
  - Outcome: food insecurity
  - Predictor: perceived neighborhood availability of fruit and vegetables
  - Adjusted for SES, age and gender identity


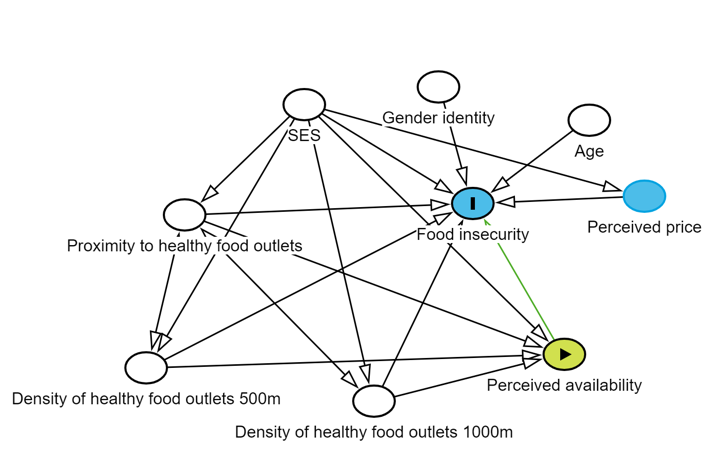


- Model 12:
  - Outcome: food insecurity
  - Predictor: perceived price of fruit and vegetables in the neighborhood
  - Adjusted for money spend on food per week, SES, age and gender identity


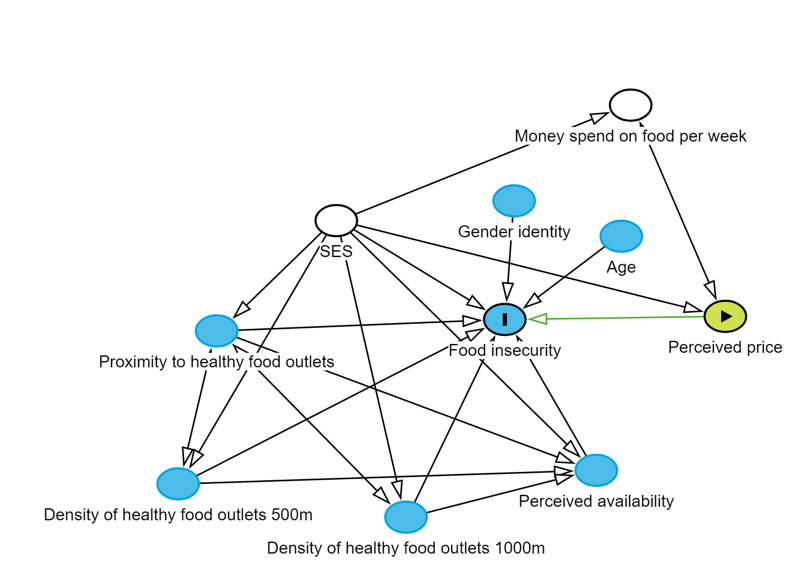


Directed Acyclic Graphs for the overall model shown in table 3:

- Overall model:
  - Outcome: food insecurity
  - Predictors: socioeconomic status, subjective health, transport to food outlets, social cohesion, social isolation, perceived neighborhood availability of fruit and vegetables, perceived price of fruit and vegetables in the neighborhood
  - Adjusted for age and gender identity


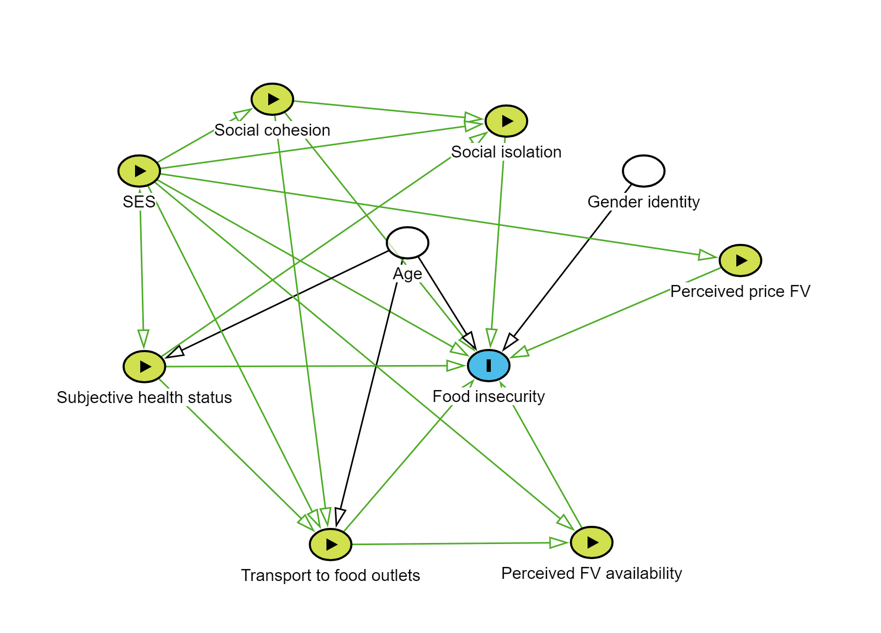

Supplement: Supplementary file 1 — Supplementary Material 1. [file 12889_2024_19389_MOESM1_ESM.docx]
